# Patterns of genetic diversity in three plant lineages endemic to the Cape Verde Islands

**DOI:** 10.1093/aobpla/plv051

**Published:** 2015-05-15

**Authors:** Maria M. Romeiras, Filipa Monteiro, M. Cristina Duarte, Hanno Schaefer, Mark Carine

**Affiliations:** 1 Biosystems and Integrative Sciences Institute (BioISI), Faculty of Sciences, University of Lisbon, Campo Grande 1749-016, Lisbon, Portugal; 2 Tropical Research Institute (IICT/JBT), Trav. Conde da Ribeira 9, 1300-142 Lisbon, Portugal; 3 Centre in Biodiversity and Genetic Resources (CIBIO/InBIO), University of Porto, Campus Agrário de Vairão, 4485-661 Vairão, Portugal; 4 Technische Universitaet Muenchen, Biodiversitaet der Pflanzen, D-85354 Freising, Germany; 5 Plants Division, Department of Life Sciences, Natural History Museum, Cromwell Road, London SW7 5BD, UK

**Keywords:** *Cynanchum*, DNA barcoding, *Globularia*, Macaronesian Islands, multi-island endemics (MIEs), *Umbilicus*

## Abstract

Conservation of plant diversity on islands relies on a good knowledge of the taxonomy, distribution and genetic diversity of species. In recent decades, a combination of morphology- and DNA-based approaches has become the standard for investigating island plant lineages and this has led, in some cases, to the discovery of previously overlooked diversity, including ‘cryptic species’. The flora of the Cape Verde archipelago in the North Atlantic is currently thought to comprise ∼740 vascular plant species, 92 of them endemics. Despite the fact that it is considered relatively well known, there has been a 12 % increase in the number of endemics in the last two decades. Relatively few of the Cape Verde plant lineages have been included in genetic studies so far and little is known about the patterns of diversification in the archipelago. Here we present an updated list for the endemic Cape Verde flora and analyse diversity patterns for three endemic plant lineages (*Cynanchum*, *Globularia* and *Umbilicus*) based on one nuclear (ITS) and four plastid DNA regions. In all three lineages, we find genetic variation. In *Cynanchum*, we find two distinct haplotypes with no clear geographical pattern, possibly reflecting different ploidy levels. In *Globularia* and *Umbilicus*, differentiation is evident between populations from northern and southern islands. Isolation and drift resulting from the small and fragmented distributions, coupled with the significant distances separating the northern and southern islands, could explain this pattern. Overall, our study suggests that the diversity in the endemic vascular flora of Cape Verde is higher than previously thought and further work is necessary to characterize the flora.

## Abstract (precis)

In an effort to better understand the evolution of the vascular plant flora of the Cape Verde Islands (Macaronesian Region), this study provides an updated checklist for the endemic vascular plants of the Cape Verde Islands and compares patterns of genetic diversity within three endemic plant lineages. The detected levels of genetic differentiation between islands indicate the existence of overlooked (cryptic) taxa in all three lineages, in the genus *Umbilicus* possibly at species level. These findings indicate that plant diversity in Cape Verde is higher than previously thought and highlights the need for additional studies.

## Introduction

Efforts to conserve island floras and to understand their diversity are crucially dependent on baseline taxonomic knowledge: species are the unit of conservation actions, the focus of phylogenetic and phylogeographic work and the basic units for macro-ecological analyses. However, whilst island floras have been subject to study over a long period (e.g. Romeiras *et al*. 2014), and are often considered well explored (e.g. Joppa *et al*. 2011), recent discoveries of island taxa new to science have occurred even in groups of large organisms such as lizards and birds (Whittaker and Fernández-Palacios 2007).

Gray and Cavers (2013) showed that taxonomic effort expended contributes to the patterns investigated in theoretical biogeography and a recent survey of biologists working on oceanic islands suggested that ‘Knowledge of the taxonomy, distribution and threat status of plants on oceanic islands is insufficient’ (Caujapé-Castells *et al*. 2010). Clearly, the taxonomy of oceanic island plants is far from complete, and this is an important issue to address.

**Figure 1.**
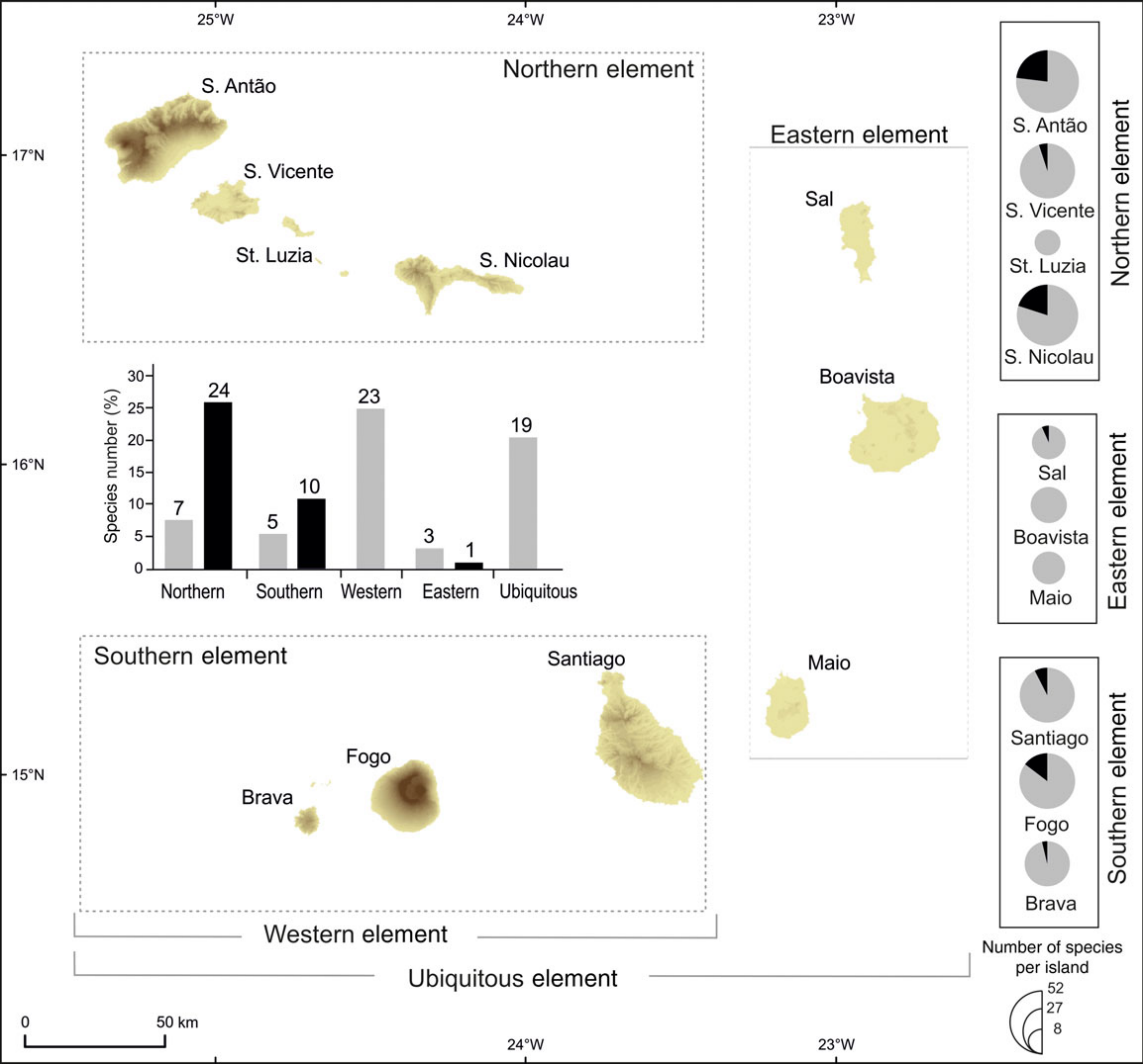
Relationship between the vascular endemic species of Cape Verde and their distribution within the five distributional elements: northern (i.e. Santo Antão, São Vicente, Santa Luzia and São Nicolau), southern (i.e. Santiago, Fogo and Brava), western (including species simultaneously present in northern and southern islands), eastern (i.e. Maio, Sal and Boavista) and ubiquitous (including species present in both western and eastern islands). The distribution of multi-island endemics—MIEs (grey) and single-island endemics—SIEs (black) in each island (right); and within the five distributional elements (bar graph in the centre; the number of taxa is placed above each bar).

This paper considers recent developments in our understanding of the flora of the Cape Verde Islands and the potential of molecular data to provide further insights. The Cape Verdes are the most southerly archipelago of the Macaronesian Region that also comprises the archipelagos of the Azores, Canaries and Madeira in the North Atlantic. The Cape Verdes are a group of 10 volcanic islands located 1500 km southwest of the Canary Islands and ∼570 km west of the African mainland. Lying at tropical latitudes and in close proximity to the coast of Senegal, their flora is of mainly tropical African origin and, accordingly, it was proposed to group the Cape Verde flora with the palaeotropical floras of the Saharan Tropical region (Rivas-Martínez 2009). Within the archipelago, the islands form three clusters: (i) northern group (Santo Antão, São Vicente, Santa Luzia and São Nicolau); (ii) southern group (Santiago, Fogo and Brava) and (iii) eastern group (Sal, Boavista and Maio) (see Fig. 1). The northern and the southern islands are characterized by high mountains [e.g. Monte Gordo (1304 m) in São Nicolau; Pico da Antónia (1392 m) in Santiago; Tope de Coroa (1979 m) in Santo Antão and Pico do Fogo (2829 m) in Fogo], offering a wide range of habitats over relatively short distances (Duarte and Romeiras 2009). The eastern islands are lower, drier and more homogeneous in their ecology. The islands’ ages range from ∼25.6 to ∼21.1 Ma (for Sal and Maio, respectively) to <6 Ma (for Brava), with ages decreasing from east to west (Doucelance *et al*. 2003). Only Fogo Island currently has volcanic activity, with the most recent eruptions occurring in 1995 and 2014.

Extensive taxonomic and collecting activity during the last decade of the 20th century resulted in the publication of a monograph on the Cape Verde endemic flora by Brochmann *et al*. (1997). A total of 82 endemic taxa were recognized and 5 distributional elements were identified: (i) northern; (ii) southern, which together form the (iii) western element; (iv) eastern and (v) ubiquitous, which comprises taxa distributed on at least one island of western and eastern elements (see Fig. 1). The vast majority of taxa that occur in the northern, southern and western distributional elements are montane; all eastern taxa are coastal; most of the geographically ubiquitous taxa are also ecologically ubiquitous, and more than half are also altitudinally ubiquitous.

To date, molecular analyses of the Cape Verde flora have been limited. Analyses of the relationships of Macaronesian lineages have focussed largely on the floras of the Azores, Canaries and Madeira [e.g. for reviews see Carine and Schaefer (2010), Caujapé-Castells (2011), Pérez-de-Paz and Caujapé-Castells (2013)], and where Cape Verdean taxa have been included, the sampling has often been limited (e.g. *Tornabenea*, Spalik and Downie 2007; *Lotus*, Ojeda *et al*. 2014). However, molecular studies of Cape Verdean *Campanula* L. (Alarcón *et al*. 2013) and *Echium* L. (Romeiras *et al*. 2007, 2011) have sampled more extensively and revealed the geographic structure of genetic variation within those lineages. For *Echium*, a single colonization from the Canaries ∼5 Ma was inferred, with subsequent diversification within the Cape Verdes during the Pleistocene (<1.8 Ma) and a split between the ‘southern’ (*E. hypertropicum*, *E. vulcanorum*) and ‘northern’ (*E. stenosiphon* s.l.) island species (García-Maroto *et al*. 2009; Romeiras *et al*. 2011). Within *Campanula*, Alarcón *et al*. (2013) inferred a recent divergence (1.0 Ma) of the Cape Verde endemic *C. jacobaea* from its sister species *C. balfourii* (endemic to Socotra). Within the archipelago, three groups were identified: two restricted to the northern islands (one each endemic to São Nicolau and Santo Antão) and a third restricted to the southern islands (Fogo, Santiago and Brava). Thus, both Romeiras *et al*. (2011) and Alarcón *et al*. (2013) found molecular patterns within lineages that are consistent with the distributional elements previously recognized by Brochmann *et al*. (1997) based on analyses of distributional data.

This paper has two goals. First, we provide a revised list of Cape Verde endemic taxa, discuss the new species discoveries and re-evaluate the distributional elements defined by Brochmann *et al*. (1997) in light of endemic taxa and distribution data published since 1997. Second, we investigate the patterns of genetic variation within three plant lineages endemic to Cape Verde to explore the geographical structuring of genetic variation in the highly fragmented insular landscape of this archipelago. We chose *Globularia* L., *Cynanchum* L. and *Umbilicus* DC. For each of these lineages, only one species is currently recognized in the Cape Verdes: *Globularia amygdalifolia* Webb (Plantaginaceae), *Cynanchum daltonii* (Decne. ex Webb) Liede & Meve (Apocynaceae) and *Umbilicus schmidtii* Bolle (Crassulaceae). *Globularia amygdalifolia* and *U. schmidtii* constitute western elements *sensu* Brochmann *et al*. (1997) with distributions spanning five and four islands, respectively. *Cynanchum daltonii*, considered a ubiquitous element, occurs on seven islands in a variety of habitats from sea level to high mountain areas. The three taxa also differ in their phytogeographic relationships: *G. amygdalifolia* has Canaro-Madeiran affinities (a Macaronesian element), *C. daltonii* Sudano–Zambesian–Sindian affinities (an African element) and *U. schmidtii* is a Mediterranean element (Brochmann *et al*. 1997). Sampling multiple accessions from across the distribution range of each species for both nuclear (ITS) and plastid DNA regions (*matK*, *psbA-trnH*, *rbcL*, *trnL-F*), we aim to determine whether a molecular variation in the three focal lineages correlates with geographic distance.

## Methods

### Characterization of the endemic flora

We compiled a list of the Cape Verde endemic vascular plants, including the recently described species, together with species distribution data, arranged according to the five distributional elements established by Brochmann *et al*. (1997): northern, southern, western (including species simultaneously present in northern and southern islands), eastern and ubiquitous (including species present in both western and eastern islands) (Fig. 1). Each species was also characterized as either hygrophytic, mesophytic or xerophytic following Brochmann *et al*. (1997). We determined the occurrence of single-island endemics (SIEs) and multi-island endemics (MIEs) for each distributional element and ecological class.

### Eco-geographic data of studied plant lineages

*Globularia amygdalifolia* and *Umbilicus schmidtii* occur in small populations of few individuals in northeast-exposed areas over 400 m above sea level (a.s.l.). *Umbilicus schmidtii* populations are found in humid zones mainly associated with montane rupicolous vegetation and *Globularia amygdalifolia* is mainly found in montane scrub vegetation, remarking its presence in volcanic lapilli areas of Fogo Island. *Cynachum daltonii* mainly occurs in semi-arid rocky escarpments up to 900 m a.s.l., reaching more than 2000 m a.s.l. on Fogo. It is a characteristic component of the northeast-exposed coastal cliffs, where it forms large stands.

### Molecular analyses

#### Sampling

Plant material for DNA extraction was collected and preserved in silica gel. Vouchers were deposited at LISC (Tropical Research Institute) and BM (Natural History Museum, London) herbaria [**see Supporting Information**].

*Globularia amygdalifolia* populations were sampled from four of the five islands on which it occurs: São Nicolau (one population, five individuals), Santo Antão (one population, two individuals), Fogo (two populations, five individuals) and Brava (two populations, five individuals) [**see Supporting Information**]. We did not sample plants from Santiago, since all individuals seen were cultivated.

For *Cynachum daltonii*, populations were sampled on all seven islands from which it has been recorded but DNA extraction from the samples from Santiago and São Vicente failed. The included samples are: São Nicolau (three individuals), Santo Antão (five individuals), Boavista (three individuals), Fogo (three individuals) and Brava (five individuals) with one population sampled on each island except Brava where we sampled two populations [**see Supporting Information**].

For *Umbilicus schmidtii*, individuals were sampled from all four islands on which it occurs: São Nicolau (one population, five individuals), Santo Antão (three populations, five individuals), Santiago (one population, five individuals) and Fogo (two populations, five individuals).

#### Molecular methods

DNA extraction from silica gel-dried leaf material followed a DNeasy Plant Mini Kit protocol (Qiagen, Crawley, UK), with a further purification using QIAquick PCR Purification Kit (Qiagen, Crawley, UK).

One individual per island per species was selected for a first molecular screening with five DNA regions [i.e. ITS and four cpDNA (*matK*, *psbA-trnH*, *rbcL*, *trnL-F*)] in order to ascertain which regions are variable within each species. Thereafter, only variable regions were amplified and sequenced for the remaining samples.

From the nuclear genome, the ITS1–5.8S–ITS2 region was amplified using ITS5 and ITS4 primers from White *et al*. (1990). From the chloroplast genome, four regions were sequenced: parts of the maturase K (*matK*) gene using the primers *matKF*_uni: 5′-AAT TTA CGA TCH ATT CAT TCM ATW TTT CC-3′ and *matKR*_uni: 5′-AGT TYT ARC ACA AGA AAG TCG AAR TAT ATA-3′ following Schaefer *et al*. (2011); the ribulose-1,5-bisphosphate carboxylase/oxygenase (*rbcL*) gene using the primers 1F and 724R of Olmstead *et al*. (1992); the *trnL*-*F* spacer using the primers ‘e’ and ‘f’ of Taberlet *et al*. (1991) and the *psbA-trnH* spacer using the primers of Sang *et al*. (1997). DNA amplification was performed in a 2720 Thermal Cycler (Applied Biosystems) in 25 μL-volume reactions. Standard polymerase chain reaction (PCR) procedures were applied to carry out amplifications. We used 1.25 units of DreamTaq™ DNA polymerase, and BSA (0.4 mg mL^−1^) for all reactions. The PCR conditions were as follows: (i) 10 min pre-treatment at 94 °C, 28 cycles of 1 min at 95 °C, 1 min at 55 °C, 3 min at 72 °C and a final stage of 7 min at 72 °C, for ITS; (ii) 10 min pre-treatment at 94 °C, 30 cycles of 1 min at 96 °C, 3 min at 50 °C, 3 min at 72 °C and a final stage of 7 min at 72 °C, for the cpDNA region. Amplified products were purified with Sureclean Plus (Bioline, London, UK) and sent to STAB Vida—Investigação e Serviços em Ciências Biológicas, Lda (Monte da Caparica, Portugal) for Sanger sequencing. Sequences were deposited in GenBank under the accession numbers KP279325–KP279464 [**see Supporting Information**].

Raw sequences were edited with BioEdit v.7.0.9 (Hall 1999) and alignments were performed in ClustalX v.2.0.10 (Thompson *et al*. 1997) using default parameters. Chloroplast DNA regions were concatenated using Sequence Matrix 1.7.8 (Vaidya *et al*. 2011). Gaps and inversions were coded as single mutations and a statistical-parsimony network (Templeton *et al*. 1992) was constructed using TCS vers. 1.21 (Clement *et al*. 2000) with a 95 % parsimony criterion.

## Results

### Characterization of the endemic flora

Ninety-two taxa (including several subspecies) are currently considered endemic to the Cape Verde Islands (Table 1). The majority of endemic taxa (∼75 %) are distributed in the western islands, either in the (i) northern (34 %), (ii) southern (16 %) elements or in the (iii) western element (25 %); lower proportions are distributed in the eastern islands (4 %) or are ubiquitous elements (21 %) (Fig. 1 and Table 1). The northern islands are also the richest in SIEs (Fig. 1). Twenty-four of the 31 taxa that constitute the northern element are SIEs (77 %). The remaining seven taxa of the northern element occur on two to three islands with none found on Santa Luzia (Table 1). Ten SIEs occur in the southern element (six SIEs in Fogo, three in Santiago and one in Brava), constituting 66 % of the southern element. Of the four eastern element taxa, only one is a SIE (25 %).

**Table 1.** List of the endemic vascular plants of Cape Verde grouped by distributional elements (*sensu* Brochmann *et al*. 1997). The acronyms for each island are as follows: SA, Santo Antão; SV, São Vicente; SL, Santa Luzia; SN, São Nicolau; B, Boavista; M, Maio; ST, Santiago; F, Fogo and BR, Brava.

Distributional elements / Taxon	SA	SV	SL	SN	S	B	M	ST	F	Br	Ecological groups
*Northern element*											
*Aeonium gorgoneum* J. A. Schmidt	X	X		X							Mesophytic
*Asteriscus smithii* (Webb) Walp.				X							Hygrophytic
*Campylanthus glaber* Benth. subsp. *spathulatus* (A. Chev.) Brochmann, N. Kilian, Lobin & Rustan	X										Mesophytic
*Carex antoniensis* A. Chev.	X										Hygrophytic
*Carex paniculata* L. subsp. *hansenii* Lewej. & Lobin	X										Hygrophytic
*Conyza schlechtendalii* Bolle				X							Hygrophytic
*Diplotaxis antoniensis* Rustan	X										Xerophytic
*Diplotaxis gorgadensis* Rustan subsp. *brochmannii* Rustan	X										Hygrophytic
*Diplotaxis gorgadensis* Rustan subsp. *gorgadensis*	X										Mesophytic
*Diplotaxis gracilis* (Webb) O. E. Schulz				X							Mesophytic
*Diplotaxis sundingii* Rustan				X							Hygrophytic
*Diplotaxis vogelli* (Webb) Cout.		X									Mesophytic
*Echium stenosiphon* Webb subsp. *glabrescens* (Pett.) Romeiras & Maria C. Duarte				X							Mesophytic
*Echium stenosiphon* Webb subsp. *lindbergii* (Pett.) Bramwell	X										Mesophytic
*Echium stenosiphon* Webb subsp. *stenosiphon*		X									Mesophytic
*Frankenia ericifolia* Chr. Sm. ex DC. subsp. *caboverdeana* Brochmann, Lobin & Sunding	X	X		X							Mesophytic
*Frankenia ericifolia* Chr. Sm. ex DC. subsp. *montana* Brochmann, Lobin & Sunding				X							Hygrophytic
*Helichrysum nicolai* N. Kilian, Galbany & Oberpr.				X							Mesophytic
*Kickxia elegans* (G. Forst.) D. A. Sutton subsp. *webbiana* (Sunding) Rustan & Brochmann	X										Mesophytic
*Launaea gorgadensis* (Bolle) N. Kilian	X	X		X							Mesophytic
*Launaea picridioides* (Webb) Engler	X	X		X							Mesophytic
*Limonium jovi-barba* (Webb) Kuntze		X		X							Hygrophytic
*Limonium sundingii* Leyens, Lobin, N. Kilian & Erben				X							Hygrophytic
*Lobularia canariensis* (DC.) Borgen subsp. *spathulata* (J. A. Schmidt) Borgen		X		X							Mesophytic
*Lotus alianus* J.H. Kirkbr.	X	X									Xerophytic
*Lotus arborescens* Lowe ex Cout.				X							Hygrophytic
*Lotus oliveirae* A. Chev.	X										Mesophytic
*Papaver gorgoneum* Cout. subsp. *theresias* Kadereit & Lobin	X										Mesophytic
*Teline stenopetala* (Webb & Berthel.) Webb & Berthel. subsp. *santoantaoi* Marrero-Rodr.	X										Hygrophytic
*Tornabenea bischoffii* J. A. Schmidt	X										Hygrophytic
*Tornabenea ribeirensis* Schmidt & Lobin				X							Mesophytic
*Southern element*											
*Asteriscus daltonii* (Webb) Walp. subsp. *daltonii*								X			Mesophytic
*Campanula bravensis* (Bolle) A. Chev.								X	X	X	Hygrophytic
*Centaurium tenuiflorum* (Hoffmanns. & Link) Fritsch subsp. *viridense* (Bolle) A. Hansen & Sunding								X	X	X	Hygrophytic
*Diplotaxis hirta* (A. Chev.) Rustan & Borgen									X		Mesophytic
*Diplotaxis varia* Rustan								X		X	Mesophytic
*Echium hypertropicum* Webb								X		X	Mesophytic
*Echium vulcanorum* A. Chev.									X		Mesophytic
*Erysimum caboverdeanum* (A. Chev.) Sund.									X		Mesophytic
*Launaea thalassica* N. Kilian, Brochmann & Rustan										X	Mesophytic
*Limonium lobinii* N. Kilian & T. Leyens								X			Hygrophytic
*Lotus jacobaeus* L.								X	X		Mesophytic
*Tornabenea annua* Bég.								X			Hygrophytic
*Tornabenea humilis* Lobin & K. H. Schmidt									X		Mesophytic
*Tornabenea tenuissima* (A. Chev.) A. Hans. & Sunding									X		Hygrophytic
*Verbascum cystolithicum* (B. Petterson) Huber-Morath									X		Mesophytic
*Western element* (northern and southern elements)											
*Artemisia gorgonum* Webb	X							X	X		Mesophytic
*Campanula jacobaea* C. Sm. ex Webb	X	X		X				X			Mesophytic
*Campylanthus glaber* Benth. subsp. *glaber*	X	X		X				X	X	X	Mesophytic
*Conyza feae* (Bég.) Wild	X	X		X				X	X	X	Mesophytic
*Conyza pannosa* Webb	X	X		X				X		X	Hygrophytic
*Conyza varia* (Webb) Wild	X	X		X					X	X	Mesophytic
*Dracaena draco* (L.) L. subsp. c*aboverdeana* Marrero-Rodr. & R. Almeida	X	X		X				X	X	X	Mesophytic
*Dryopteris gorgonea* J.P. Roux	X	X		X					X		Hygrophytic
*Eragrostis conerti* Lobin	X	X		X				X	X		Hygrophytic
*Globularia amygdalifolia* Webb	X			X				X	X	X	Mesophytic
*Helianthemum gorgoneum* Webb	X		X						X	X	Mesophytic
*Kickxia elegans* (G. Forst.) D. A. Sutton subsp. *dichondrifolia* (Benth.) Rustan & Brochmann	X	X		X				X			Hygrophytic
*Lavandula rotundifolia* Benth.	X	X		X				X	X		Mesophytic
*Limonium braunii* (Bolle) A. Chev.	X			X					X	X	Mesophytic
*Lobularia canariensis* (DC.) Borgen subsp. *fruticosa* (Webb) Borgen	X			X				X	X	X	Mesophytic
*Micromeria forbesii* Benth.	X			X				X	X	X	Mesophytic
*Papaver gorgoneum* Cout. subsp. *gorgoneum*				X					X		Hygrophytic
*Periploca chevalieri* Browicz	X			X				X	X	X	Mesophytic
*Phagnalon melanoleucum* Webb	X	X		X				X	X		Hygrophytic
*Sonchus daltonii* Webb	X	X		X				X	X		Hygrophytic
*Tolpis farinulosa* (Webb) Schmidt	X	X						X	X	X	Hygrophytic
*Tornabenea insularis* (Parl. ex Webb) Parl. ex Webb		X		X						X	Hygrophytic
*Umbilicus schmidtii* Bolle	X			X				X	X		Hygrophytic
*Eastern element*											
*Diplotaxis glauca* J. A. Schmidt					X	X					Xerophytic
*Fagonia mayana* Schlecht.						X	X				Xerophytic
*Pulicaria burchardii* Hutch. subsp. *longifolia* Gamal-Eldin					X						Xerophytic
*Sporobolus minutus* Link subsp. *confertus* (J. A. Schmidt) Lobin, N. Kilian & Leyens					X		X				Xerophytic
*Ubiquitous element* (northern, southern and eastern elements)											
*Aristida cardosoi* Cout.	X	X	X	X	X	X	X	X	X	X	Xerophytic
*Asparagus squarrosus* J. A. Schmidt	X	X	X	X	X	X	X				Xerophytic
*Asteriscus daltonii* subsp. *vogelii* (Webb) Greuter	X	X		X			X	X	X	X	Mesophytic
*Brachiaria lata* (Schumach.) C. E. Hubb. subsp. *caboverdeana* Conert & Ch. Köhler		X		X		X		X			Xerophytic
*Cynanchum daltonii* (Decne. ex Webb) Liede & Meve	X	X		X		X		X	X	X	Xerophytic
*Euphorbia tuckeyana* Steud. ex Webb	X	X	X	X	X	X		X	X	X	Mesophytic
*Forsskaolea procridifolia* Webb	X	X	X	X	X		X	X	X	X	Xerophytic
*Kickxia elegans* (G. Forst.) D. A. Sutton subsp. *elegans*	X	X		X	X	X	X	X	X	X	Xerophytic
*Limonium brunneri* (Webb) Kuntze		X	X		X						Xerophytic
*Lotus brunneri* Webb		X	X		X	X	X				Xerophytic
*Lotus purpureus* Webb	X	X		X		X		X	X	X	Xerophytic
*Paronychia illecebroides* Webb	X	X	X	X		X	X	X	X		Xerophytic
*Phoenix atlantica* A. Chev.					X	X	X	X			Xerophytic
*Polycarpaea gayi* Webb	X	X		X	X			X	X		Mesophytic
*Pulicaria diffusa* (Shuttlew. ex Brunn.) Pett.					X	X	X	X	X		Xerophytic
*Sideroxylon marginata* (Decne.) Cout.	X	X		X	X	X		X	X	X	Mesophytic
*Solanum rigidum* Lam.	X	X		X		X	X	X	X	X	Mesophytic
*Verbascum capitis-viridis* Hub.- Mor.	X	X		X		X	X	X			Mesophytic
*Withania chevalieri* A.E. Gonç.	X	X			X				X		Xerophytic

Nineteen endemic taxa (SIEs + MIEs) are xerophytes (21 %), 46 are mesophytes (50 %) and 27 (29 %) are hygrophytes. Hygrophytes and mesophytic endemics are most prevalent in the northern, southern and western elements: of the 69 endemic taxa that constitute these three elements, only 2 (3 %) are xerophytes; 40 are mesophytes (58 %) and 27 (39 %) are hygrophytes. All four eastern island endemics are xerophytes. The ubiquitous endemics are mainly xerophytes (13 taxa, 68 %) with the remaining six taxa considered mesophytes. Among the 35 taxa that are SIEs in Cape Verde, 54 % are mesophytes and 40 % hygrophytes and 6 % xerophytes (Table 1).

### Molecular analyses

**Figure 2.**
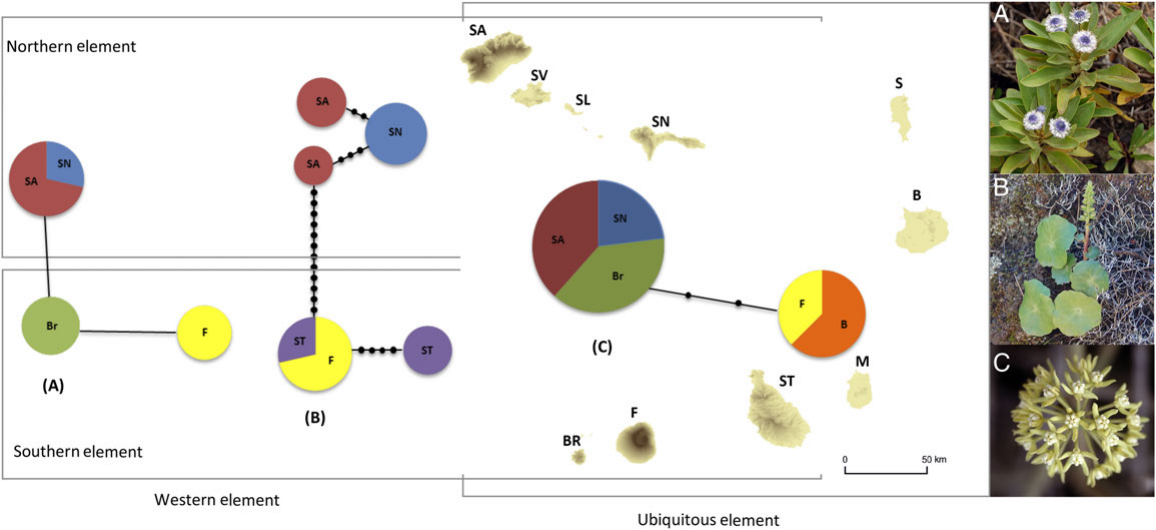
Distribution elements and haplotype network for three plant lineages produced with TCS 1.21: (A) *Globularia amygdalifolia* based on ITS; (B) *Umbilicus schmidtii* based on cpDNA (*matK*, *psbA-trnH* and *trnL-F*); (C) *Cynanchum daltonii* based on ITS. The size of circles representing each haplotype is proportional to the number of individuals possessing that haplotype. The acronyms for each island are as follows: SA, Santo Antão; SV, São Vicente; Sl, Santa Luzia; SN, São Nicolau; S, Sal; B, Boavista; M, Maio; ST, Santiago; F, Fogo; and BR, Brava. Plant species (flowering) on the right (photos M.R. and M.C.D.).

*Globularia amygdalifolia*—genetic variation was detected only in the ITS region. Two substitutions in the 659 bp ITS fragment defined three ribotypes, one of which was found only in plants from Fogo, the second restricted to Brava and the third shared between plants from the northern islands of Santo Antão and São Nicolau (Fig. 2A). The network supported a split between plants from the northern and southern islands.

*Umbilicus schmidtii*—genetic variation was observed in the *matK* (844 bp), *psbA-trnH* (289 bp) and *trnL-F* spacer (286 bp) regions (the ITS region was not successfully sequenced for most samples). A total of 11 substitutions, 9 indels and 1 inversion (8 bp) defined five haplotypes for the combined 1420 bp of plastid DNA. Three haplotypes were private to the northern island populations; of which two were found in plants from Santo Antão (one comprising plants from Cova and the other comprising plants from Pedra Rachada/Delgadinho da Corda) and one was found in São Nicolau. Three to four mutations separated the three northern island haplotypes. The two remaining haplotypes were found in plants from the southern islands. Both haplotypes occurred in the population on Santiago with one also found in Fogo. The two southern haplotypes are distinguished by 5 mutations with 13 mutations separating southern and northern haplotype groups (Fig. 2B).

*Cynanchum daltonii*—genetic variation was detected only in the ITS region: three substitutions were found in the 648 bp fragment and these defined two ribotypes (Fig. 2C). One was found in plants from Santo Antão, São Nicolau and Brava, and the second in plants from Fogo and Boavista.

## Discussion

### Spatial patterns of endemism

The endemic vascular plant list for Cape Verde has increased from 82 (Brochmann *et al*. 1997) to 92 taxa, which is an ∼12 % increase in 18 years. Of the endemics more recently discovered or reclassified as endemics, one is a fern (*Dryopteris gorgonea*) and the remainder are angiosperms, of which six are recognized at species rank (*Fagonia mayana*, *Helichrysum nicolai*, *Lotus alianus*, *Tornabenea ribeirensis*, *Solanum rigidum* and *Withania chevalieri*) and three at subspecies rank (*Echium stenosiphon* subsp. *glabrescens*, *Teline stenopetala* subsp. *santoantonai* and *Dracaena draco* subsp. *caboverdeana*). Three of the 10 (*Echium stenosiphon* subsp. *glabrescens*, *Lotus alianus* and *Tornabenea ribeirensis*) have other conspecific Cape Verdean endemic taxa (Table 1) and their discovery reflects an enhanced understanding of lineages that have diversified within the Cape Verdes. The remaining taxa lack conspecific endemic taxa and may be considered anagenetic lineages *sensu* Stuessy *et al*. (2006). Their description has typically occurred within the context of wider revisionary or monographic studies. *Solanum rigidum*, for example, was previously considered introduced to the Cape Verdes from the Americas (Gonçalves 2002 sub *S. fuscatum* L.). However, monographic research on the genus has shown it to be a Cape Verde endemic that has rather been introduced into the Americas (Knapp and Vorontsova 2013). The discovery of the anagenetic element of the Cape Verde endemic flora is likely far from complete. Whilst many such taxa are widespread taxa of little conservation concern, this is not the case for all. For example, *Helichrysum nicolai*, described by Kilian *et al*. (2010), is restricted to the Alto das Cabaças range in the northeastern part of São Nicolau. Its conservation status has not been assessed, but population sizes (very few tens) and the extent and area of occurrence would all appear to be extremely limited, suggesting a high threat status.

The overall patterns in the endemic flora recognized by Brochmann *et al*. (1997) are still evident in the updated list of endemics presented in Table 1. Thus, the five distributional elements are clearly distinguished. Three quarters of endemic taxa are restricted to the western islands, nearly all of which are mesophytes or hygrophytes and a high proportion are SIEs. In general, populations of both SIEs and MIEs in the northern, southern and western elements are small, and restricted to specific habitats in N–NE facing moist cliffs of mountain areas (Duarte *et al*. 2008). Their distributions are thus highly fragmented, and this is likely to have led to isolation and drift resulting in the high incidence of single-island endemism observed. Xerophytes dominate among eastern island endemics and the ubiquitous element wherein 68 % are xerophytes. Indeed, only one SIE is a xerophyte. The generally larger population sizes of lowland xerophytic taxa coupled with the relatively close proximity of islands (e.g. only 15 km separates the coastal areas of Santo Antão from São Vicente, in the northern islands) may facilitate gene flow between islands, maintaining the integrity of these taxa.

### Genetic diversification within the plant lineages

The molecular analyses we present in this paper provide further insights into the patterns of endemic plant diversity in the Cape Verdes. *Globularia amygdalifolia* and *Umbilicus schmidtii* both constitute western elements of the flora (Table 1) and in both cases, differentiation is evident at the molecular level between plants from southern and northern islands (see Fig. 2A and B). Isolation and drift resulting from the small and fragmented distributions of these taxa coupled with the significant distances separating the northern and southern sub-archipelagos (230 km separates Santo Antão from Brava; and 140 km São Nicolau from Santiago, maximum and minimum distances, respectively) is likely to explain this pattern. A similar intraspecific pattern was documented in *Campanula jacobaea* (Alarcón *et al*. 2013), which is also a western element. From a conservation perspective, it would be appropriate to treat northern and southern populations of western element taxa as distinct management units.

In the case of *U. schmidtii*, the differentiation of northern and southern populations appears to be consistent with differences observed in the inflorescence (e.g. morphological characters of the flowers, data not shown) suggesting the need of further research, in order to ascertain whether overlooked taxa occur in the archipelago. *Umbilicus* is a small succulent herb, which is perhaps most likely to harbour cryptic diversity because it is an undercollected species, and most of the herbarium specimens lack important characters for identification. Its tiny seeds are probably wind-dispersed, but gene flow might be reduced due to the significant distance between the islands.

Some variation in leaf size and shape was also evident within *G. amygdalifolia*, but no clear geographical pattern in the morphological variation could be identified. Nevertheless, a morphological re-examination in this species within the context of the molecular results presented here would be appropriate.

Intraspecific variation was also found in *C. daltonii* with two distinct haplotypes. However, the distribution of the two haplotypes is incongruent with Brochmann *et al*.'s (1997) elements since one is distributed in the eastern island of Boavista and the southern island of Fogo whereas the other is distributed in the northern islands of Santo Antão and São Nicolau and the southern island of Brava. Further sampling including Santiago and São Vicente is needed to better understand the patterns of genetic diversity in this species. An analysis of ploidy level of plants might also be informative since two ploidy levels have been provisionally reported for *C. daltonii* (Brochmann *et al*. 1997; Meve and Liede-Schumann 2012) and this may explain the occurrence of the two distinct haplotypes.

Our study has surveyed three plant lineages and it would be premature to make generalizations regarding the entire flora. Nevertheless, it does appear that levels of intraspecific genetic diversity could be similar to that reported from the Azores Islands (Schaefer *et al*. 2011; Rumsey *et al*. 2014). Although there are more SIEs in the Cape Verdes than in the Azores, a high proportion of MIE relative to SIE is reported for both archipelagos in comparison with the Canaries and Madeira (Carine and Schaefer 2010). In both cases, plant lineages that are widespread across the archipelago and that exhibit little morphological diversification have nevertheless been found to exhibit geographically structured molecular patterns.

Oceanic islands such as the Cape Verdes have been the focus of taxonomic research during the last 200 years (Romeiras *et al*. 2014), but our baseline taxonomic knowledge still needs to be refined to effectively support conservation and provide a context for understanding the evolution and biogeography of island plants. Knowledge of the endemic flora has developed significantly in the last two decades but our molecular results suggest that further taxonomic revisions, integrating genetic, morphological and ecological data, among others, are still necessary to better understand the patterns of diversity in the endemic flora. Widespread species, in particular, should be the focus of taxonomic work since they may contain overlooked taxa, and species as they are currently circumscribed, may not be the most appropriate management unit from a conservation perspective.

## Sources of Funding

This work was supported by the Portuguese Foundation for Science and Technology (FCT) and European Social Funds through project PTDC/BIA-BIC/4113/2012.

## Contributions by the Authors

M.R. and M.C. conceived the study. M.R., M.C.D. and M.C. conducted the sampling in Cape Verde. M.R. and F.M. carried out molecular work and M.C.D. carried out morphological observations. All authors analyzed data. M.R. wrote the first draft: M.C. and H.S. improved upon versions. All authors read and approved the final manuscript.

## Conflict of Interest Statement

None declared.

## Supplementary Material

Additional Information

## Supporting Information

The following additional information is available in the online version of this article –

**Table S1.** Taxon sampling, GenBank accessions numbers and voucher specimens.
