# Supplementary material for: Patterns of genetic diversity in three plant lineages endemic to the Cape Verde Islands
Source: AoB Plants. 2015 May 15;7:plv051. doi: 10.1093/aobpla/plv051 (PMC4501515; doi:10.1093/aobpla/plv051)
Supplement: Additional Information [file supp_7_plv051_index.html]

Additional Information 

# Patterns of genetic diversity in three plant lineages endemic to the Cape Verde Islands

## Additional Information

Additional Information

- Additional Information - Docx file
