# Supplementary material for: Patterns of genetic diversity in three plant lineages endemic to the Cape Verde Islands
Source: AoB Plants. 2015 May 15;7:plv051. doi: 10.1093/aobpla/plv051 (PMC4501515; doi:10.1093/aobpla/plv051)
Supplement: Additional Information [file supp_plv051_plv051supp.docx]

**Table S1.** Taxon sampling, GenBank accessions numbers and voucher specimens.

| **Island** | **Sampling Site** | **Y_Lat** | | **X_Long** | | ***ITS*** | ***matK*** | ***rbcL*** | ***psbA-trnH*** | ***trnL-F*** | **Voucher** |
| --- | --- | --- | --- | --- | --- | --- | --- | --- | --- | --- | --- |
| ***Globularia amygdalifolia*** | |  |  | |  | |  |  |  |  |  |
| **São Nicolau** | Monte Gordo, Rib. Calhaus | 16,629200 | | -24,356450 | | KP279396 |  |  |  |  | Duarte et al. 4122a, LISC |
| **São Nicolau** | Monte Gordo, Rib. Calhaus | 16,629200 | | -24,356450 | | KP279397 |  |  |  |  | Duarte et al. 4122b, LISC |
| **São Nicolau** | Monte Gordo, Rib. Calhaus | 16,629200 | | -24,356450 | | KP279398 |  |  |  |  | Duarte et al. 4122c, LISC |
| **São Nicolau** | Monte Gordo, Rib. Calhaus | 16,629200 | | -24,356450 | | KP279399 | KP279367 | KP279354 | KP279436 | KP279325 | Duarte et al. 4122d, LISC |
| **São Nicolau** | Monte Gordo, Rib. Calhaus | 16,629200 | | -24,356450 | | KP279400 |  |  |  |  | Duarte et al. 4122e, LISC |
| **Santo Antão** | Morro do Vento | 17,099317 | | -25,073983 | | KP279401 | KP279368 | KP279355 | KP279437 | KP279326 | M. Romeiras & M. Carine 2053, BM |
| **Santo Antão** | Morro do Vento | 17,099392 | | -25,073917 | | KP279402 |  |  |  |  | M. Romeiras & M. Carine 2055, BM |
| **Fogo** | Bordeira - Cave | 14,913756 | | -24,350547 | | KP279403 | KP279369 | KP279356 | KP279438 | KP279327 | M. Romeiras & M. Carine 1054, LISC |
| **Fogo** | Bordeira | 14,918300 | | -24,349975 | | KP279404 |  |  |  |  | M. Romeiras et al. 902, LISC |
| **Fogo** | Rib. Bangaeira, to Monte Velha | 14,980400 | | -24,370828 | | KP279405 |  |  |  |  | M. Romeiras et al. 806, LISC |
| **Fogo** | Rib. Bangaeira, to Monte Velha | 14,980447 | | -24,370867 | | KP279406 |  |  |  |  | M. Romeiras et al. 780, LISC |
| **Fogo** | Rib. Bangaeira, to Monte Velha | 14,980447 | | -24,370867 | | KP279407 |  |  |  |  | M. Romeiras et al. 781, LISC |
| **Brava** | Fajã d'Água | 14,868444 | | -24,722669 | | KP279408 | KP279370 | KP279357 | KP279439 | KP279328 | JC Costa & I Gomes 38/2014, LISC |
| **Brava** | Fajã d'Água | 14,868444 | | -24,722669 | | KP279409 |  |  |  |  | JC Costa & I Gomes 38/2014, LISC |
| **Brava** | Fajã d'Água | 14,868444 | | -24,722669 | | KP279410 |  |  |  |  | JC Costa & I Gomes 38/2014, LISC |
| **Brava** | Figueiral Grande | 14,868025 | | -24,721289 | | KP279411 |  |  |  |  | JC Costa & I Gomes 32/2014, LISC |
| **Brava** | Figueiral Grande | 14,868025 | | -24,721289 | | KP279412 |  |  |  |  | JC Costa & I Gomes 32/2015, LISC |
| ***Cynanchum* (=*Sarcostemma*) *daltonii*** | |  |  | |  | |  |  |  |  |  |
| **São Nicolau** | Near Rib. Brava | 16,619450 | | -24,323617 | | KP279413 | KP279371 | KP279358 | KP279440 | KP279329 | Duarte et al. 4177a, LISC |
| **São Nicolau** | Near Rib. Brava | 16,611117 | | -24,302783 | | KP279414 |  |  |  |  | Duarte et al. 4178a, LISC |
| **São Nicolau** | Near Rib. Brava | 16,611117 | | -24,302783 | | KP279415 |  |  |  |  | Duarte et al. 4178c, LISC |
| **Santo Antão** | Before Rib. Janela | 17,120653 | | -24,995106 | | KP279416 | KP279372 | KP279359 | KP279441 | KP279330 | M. Romeiras et al. 563, LISC |
| **Santo Antão** | Before Rib. Janela | 17,120653 | | -24,995106 | | KP279417 |  |  |  |  | M. Romeiras et al. 563, LISC |
| **Santo Antão** | Before Rib. Janela | 17,120653 | | -24,995106 | | KP279418 |  |  |  |  | M. Romeiras et al. 563, LISC |
| **Santo Antão** | Before Rib. Janela | 17,120653 | | -24,995106 | | KP279419 |  |  |  |  | M. Romeiras et al. 563, LISC |
| **Santo Antão** | Before Rib. Janela | 17,120653 | | -24,995106 | | KP279420 |  |  |  |  | M. Romeiras et al. 563, LISC |
| **Boavista** | Rocha Estância | 16,036617 | | -22,913533 | | KP279421 | KP279373 | KP279360 | KP279442 | KP279331 | Duarte et al. 4002a, LISC |
| **Boavista** | Rocha Estância | 16,036617 | | -22,913533 | | KP279422 |  |  |  |  | Duarte et al. 4002b, LISC |
| **Boavista** | Rocha Estância | 16,036617 | | -22,913533 | | KP279423 |  |  |  |  | Duarte et al. 4002c, LISC |
| **Boavista** | Rocha Estância | 16,036617 | | -22,913533 | | KP279424 |  |  |  |  | Duarte et al. 4002d, LISC |
| **Boavista** | Rocha Estância | 16,036617 | | -22,913533 | | KP279425 |  |  |  |  | Duarte et al. 4002e, LISC |
| **Fogo** | Monte Velha | 14,994431 | | -24,349467 | | KP279426 | KP279374 | KP279361 | KP279443 | KP279332 | M. Romeiras et al. 837, LISC |
| **Fogo** | Monte Velha | 14,994431 | | -24,349467 | | KP279427 |  |  |  |  | M. Romeiras et al. 838, LISC |
| **Fogo** | Monte Velha | 14,994431 | | -24,349467 | | KP279428 |  |  |  |  | M. Romeiras et al. 839, LISC |
| **Brava** | Figueiral | 14,875956 | | -24,682594 | | KP279429 |  |  |  |  | JC Costa & I Gomes 33/2014, LISC |
| **Brava** | Figueiral | 14,875956 | | -24,682594 | | KP279430 |  |  |  |  | JC Costa & I Gomes 33/2014, LISC |
| **Brava** | Figueiral | 14,875956 | | -24,682594 | | KP279431 |  |  |  |  | JC Costa & I Gomes 33/2014, LISC |
| **Brava** | Figueiral | 14,875956 | | -24,682594 | | KP279432 | KP279375 | KP279362 | KP279444 | KP279333 | JC Costa & I Gomes 33/2014, LISC |
| **Brava** | Santa Bárbara | 14,875956 | | -24,682594 | | KP279433 |  |  |  |  | Matos 5445, LISC |
| ***Umbilicus schmidtii*** | |  |  | |  | |  |  |  |  |  |
| **São Nicolau** | Monte Gordo, Rib. dos Calhaus | 16,629200 | | -24,356450 | | KP279434 | KP279376 | KP279363 | KP279445 | KP279334 | Duarte et al. 4125c, LISC |
| **São Nicolau** | Monte Gordo, Rib. dos Calhaus | 16,629200 | | -24,356450 | |  | KP279377 |  | KP279446 | KP279335 | Duarte et al. 4125a, LISC |
| **São Nicolau** | Monte Gordo, Rib. dos Calhaus | 16,629200 | | -24,356450 | |  | KP279378 |  | KP279447 | KP279336 | Duarte et al. 4124b, LISC |
| **São Nicolau** | Monte Gordo, Rib. dos Calhaus | 16,629200 | | -24,356450 | |  | KP279379 |  | KP279448 | KP279337 | Duarte et al. 4125d, LISC |
| **São Nicolau** | Monte Gordo, Rib. dos Calhaus | 16,629200 | | -24,356450 | |  | KP279380 |  | KP279449 | KP279338 | Duarte et al. 4125e, LISC |
| **São Nicolau** | Monte Gordo, Rib. dos Calhaus | 16,629200 | | -24,356450 | | KP279434 | KP279376 | KP279363 | KP279445 | KP279334 | Duarte et al. 4125c, LISC |
| **Santo Antão** | Delgadinho Corda | 17,145989 | | -25,083464 | |  | KP279381 |  | KP279450 | KP279339 | M. Romeiras & M. Carine 3170, LISC |
| **Santo Antão** | Pedra Rachada | 17,117667 | | -25,064333 | |  | KP279382 |  | KP279451 | KP279340 | M. Romeiras & M. Carine 2076, LISC |
| **Santo Antão** | Pedra Rachada | 17,117667 | | -25,064333 | |  | KP279383 | KP279364 | KP279452 | KP279341 | M. Romeiras & M. Carine 2075, LISC |
| **Santo Antão** | Cova | 17,104264 | | -25,060825 | |  | KP279384 |  | KP279453 | KP279342 | M. Romeiras 619, LISC |
| **Santo Antão** | Cova | 17,104264 | | -25,060825 | |  | KP279385 |  | KP279454 | KP279343 | M. Romeiras 620, LISC |
| **Santiago** | Serra Malagueta | 15,178097 | | -23,687356 | |  | KP279386 | KP279365 | KP279455 | KP279344 | M. Romeiras & M. Carine 1008, LISC |
| **Santiago** | Serra Malagueta | 15,178050 | | -23,687333 | |  | KP279387 |  | KP279456 | KP279345 | M. Romeiras & M. Carine 1007, LISC |
| **Santiago** | Serra Malagueta | 15,178050 | | -23,687333 | |  | KP279388 |  | KP279457 | KP279346 | Romeiras 920, LISC |
| **Santiago** | Serra Malagueta | 15,178050 | | -23,687333 | |  | KP279389 |  | KP279458 | KP279347 | Romeiras 920, LISC |
| **Santiago** | Serra Malagueta | 15,178050 | | -23,687333 | |  | KP279390 |  | KP279459 | KP279348 | Romeiras 920, LISC |
| **Fogo** | Monte Velha | 14,993811 | | -24,348986 | | KP279435 | KP279391 | KP279366 | KP279460 | KP279349 | M. Romeiras & M. Carine 1070, LISC |
| **Fogo** | Monte Velha | 14,993833 | | -24,349000 | |  | KP279392 |  | KP279461 | KP279350 | M. Romeiras & M. Carine 1068, LISC |
| **Fogo** | Monte Velha | 14,993836 | | 14,993836 | |  | KP279393 |  | KP279462 | KP279351 | M. Romeiras & M. Carine 1071, LISC |
| **Fogo** | Chã Caldeiras to Monte Velha | 14,994164 | | -24,348969 | |  | KP279394 |  | KP279463 | KP279352 | M. Romeiras & M. Carine 1067, LISC |
| **Fogo** | Monte Velha | 14,993836 | | 14,993836 | |  | KP279395 |  | KP279464 | KP279353 | M. Romeiras & M. Carine 1069, LISC |
